# Dissecting the causal relationship between household income status and genetic susceptibility to cardiovascular-related diseases: Insights from bidirectional mendelian randomization study

**DOI:** 10.1186/s12889-023-15561-7

**Published:** 2023-04-24

**Authors:** Xifeng Zheng, Yu Yang, Jianying Chen, Bing Lu

**Affiliations:** 1 Department of Cardiology, Affiliated Hospital of Guangdong Medical University, No.57 South of Renming Road, Zhanjiang, Guangdong China; 2 Department of Geriatrics, Affiliated Hospital of Guangdong Medical University, No.57 South of Renming Road, Zhanjiang, Guangdong China

**Keywords:** Household income status, Cardiovascular health, Causal relationship, Instrumental variable, Mendelian randomization study

## Abstract

**Objectives:**

Observational studies have revealed that socioeconomic status is associated with cardiovascular health. However, the potential causal effect remains unclear. Hence, we aimed to investigate the causal relationship between household income status and genetic susceptibility to cardiovascular-related diseases using a bidirectional Mendelian randomization (MR) study.

**Methods:**

An MR study based on a large-sample cohort of the European population from a publicly available genome-wide association study datasets was conducted using a random-effects inverse-variance weighting model as the main standard. Simultaneously, MR-Egger regression, weighted median, and maximum likelihood estimation were used as supplements. Sensitivity analysis, consisting of a heterogeneity test and horizontal pleiotropy test, was performed using Cochran’s Q, MR-Egger intercept, and MR-PRESSO tests to ensure the reliability of the conclusion.

**Results:**

The results suggested that higher household income tended to lower the risk of genetic susceptibility to myocardial infarction (OR: 0.503, 95% CI = 0.405–0.625, P < 0.001), hypertension (OR: 0.667, 95% CI = 0.522–0.851, P = 0.001), coronary artery disease (OR: 0.674, 95% CI = 0.509–0.893, P = 0.005), type 2 diabetes (OR: 0.642, 95% CI = 0.464–0.889, P = 0.007), heart failure (OR: 0.825, 95% CI = 0.709–0.960, P = 0.013), and ischemic stroke (OR: 0.801, 95% CI = 0.662–0.968, P = 0.022). In contrast, no association was evident with atrial fibrillation (OR: 0.970, 95% CI = 0.767–1.226, P = 0.798). The reverse MR study suggested a potentially negative trend between heart failure and household income status. A sensitivity analysis verified the reliability of the results.

**Conclusions:**

The results revealed that the population with higher household income tended to have a lower risk of genetic susceptibility to myocardial infarction and hypertension.

**Supplementary Information:**

The online version contains supplementary material available at 10.1186/s12889-023-15561-7.

## Introduction

According to a report by the World Health Organization, ischemic heart disease and stroke were the top two causes of mortality worldwide in 2019, accounting for 16% and 11% of deaths, respectively. Cardiovascular-related diseases (i.e., ischemic heart disease and stroke) are a major health threat to the aging population. In addition, they cause a trend of increasing morbidity in young individuals due to obesity, diabetes, and drug abuse. Over the past two decades, this trend has been reflected by an increasing incidence of ischemic stroke among young people in the United States, Sweden, France, and Denmark. Although cardiovascular-related diseases are highly prevalent, it is gratifying that with the continuous popularization of health education and secondary prevention using medications, the total number of deaths in developed countries declined from 2000 to 2019, especially among high-income populations [1, 2]. Furthermore, multiple observational studies have reported that people with superior socioeconomic status usually have a lower risk of morbidity in cardiovascular diseases or better prognosis [3]. In contrast, low-income populations may be exposed to higher risk or worse prognosis [4, 5]. This social phenomenon requires further research on the potential causal relationship between household income and cardiovascular-related disease morbidity. Unfortunately, reverse causation, measurement error, and potential bias are inherent disadvantages of observational studies that prevent clarification of the potential causal relationship. To the best of our knowledge, limited evidence exists on the causal relationship between household income status and cardiovascular disease-related morbidity, especially the lack of large-sample cohort studies.

Mendelian randomization (MR) is a method that applies valuable genetic variants, such as single nucleotide polymorphisms (SNPs), as instrumental variables (IVs) to evaluate the causal effects between modifiable, non-genetic exposure factors and genetic susceptibility to diseases. In the absence of randomized controlled trials (RCTs), MR studies represent an alternative strategy for causal inference because genetic variants are randomly assigned during meiosis to simulate the RCT process. Compared with traditional observational studies, the greatest advantage of MR studies is that they are less likely to be influenced by unmeasured confounding factors because genetic variants are identified at the time of conception [6, 7]. MR studies have been successfully applied to various causal relationship analyses between behavior exposure, education, socioeconomic conditions, and various diseases [8, 9]. Hence, this research aims to identify the bidirectional causal relationship between household income status and genetic susceptibility to common cardiovascular-related diseases using an MR study.

## Materials and methods

### Study design and GWAS datasets information

To achieve impartial results, an MR study depends on three fundamental assumptions: (1) the selected genetic IVs must be significantly associated with the exposure factor, (2) the IVs should be independent of potential confounders associated with exposure factors and outcomes, and (3) the IVs should affect the outcomes only through the exposure factor [6]. This study conducted the MR analysis 14 times to explore the bidirectional association between annual household income status and seven cardiovascular-related diseases.

The research is based on a large-sample cohort of the European population from publicly available genome-wide association study (GWAS) datasets. The variable genetic information involved in this study was extracted from the Integrative Epidemiology Unit (IEU) GWAS database [10] (https://gwas.mrcieu.ac.uk/), which is a publicly available GWAS summary database. Therefore, the requirement for ethical committee approval was waived. The GWAS summary dataset “average total household income before tax” represented the household income status of 397,751 samples originally from the UK biobank database. The annual household income was divided into five intervals: less than 18,000 pounds, 18,000 to 30,999 pounds, 31,000 to 51,999 pounds, 52,000 to 100,000 pounds, and greater than 100,000 pounds. In contrast, cardiovascular-related diseases were represented by coronary artery disease, myocardial infarction, heart failure, atrial fibrillation, hypertension, ischemic stroke, and type 2 diabetes, respectively. Detailed information on all the GWAS datasets is listed in Table 1. The household income GWAS dataset and GWAS datasets of cardiovascular diseases originated from different consortiums to decrease the potential bias caused by sample overlap. In addition, all GWAS datasets involved in this study included populations of European ancestry to mitigate bias from population stratification.

**Table 1:** Basic information of the GWAS datasets involved in the study

Traits	GWAS ID	Years	Population	Sample size
Exposure factor				Total sample
Household income status [11]	ukb-b-7408	2018	European	397,751
**Outcomes**				**Case/Control**
Coronary artery disease [12]	finn-b-I9_CHD	2021	European	21,012/197,780
Myocardial infarction [13]	ebi-a-GCST011365	2021	European	61,505/577,716
Heart failure [14]	ebi-a-GCST009541	2020	European	47,309/930,014
Atrial fibrillation [15]	ebi-a-GCST006414	2018	European	60,620/970,216
Hypertension [12]	finn-b-I9_HYPTENS	2021	European	55,917/162,837
Ischemic stroke [16]	ebi-a-GCST006908	2018	European	34,217/406,111
Type 2 diabetes [17]	ebi-a-GCST006867	2018	European	62,892/596,424

### Selection criteria for IVs

The IVs were single nucleotide polymorphisms (SNPs) filtered according to the three afore mentioned pivotal assumptions of the MR study. First, the SNPs were matched with a genome-wide statistical significance threshold (P < 5 × 10^− 8^). Second, the corresponding linkage disequilibrium was tested to confirm the presence of SNPs in the linkage disequilibrium state and to confirm that these SNPs were independent by trimming SNPs within a 0–10,000 kb window at a threshold of r^2^ < 0.001. Third, to evaluate the assumption that the IVs affect the outcomes only through the exposure factor, the potential phenotypes that may be relevant to the IVs were investigated by searching the human genotype-phenotype association database (PhenoScanner-V2, http://www.phenoscanner.medschl.cam.ac.uk/) [18]. Fourth, SNPs identified as IVs were further matched with those in the outcome GWAS dataset to establish genetic associations. The summary SNP-phenotype and SNP-outcome statistics were harmonized to ensure effect size alignment, and the palindromic SNPs were excluded. Finally, F-statistics (> 10) were used to evaluate the strength of the IVs to avoid the influence of weak instrumental bias [19].

### Mendelian randomization study and sensitivity analysis

The MR study was performed using a random-effects inverse-variance weighting (IVW) model [20] as the primary standard and three other models (MR-Egger regression [21], weighted median [22] and maximum likelihood [23]) as supplements to evaluate the potential causal relationship between household income status and seven common cardiovascular-related diseases. The reverse MR study evaluated the potential causal relationship between seven common cardiovascular-related diseases and household income status using the same methods. In addition, sensitivity analysis was performed to measure the reliability and stability of the conclusion. The sensitivity analysis consisted of (1) Cochran’s Q test (according to the IVW model or MR-Egger regression model), (2) horizontal pleiotropy test using MR-Egger intercept [24] and MR-PRESSO test [25], and (3) “leave-one-out” test (each SNP was abandoned successively to repeat the IVW analysis to identify whether any specific SNP drives the causal relationship estimate). The results are reported as odds ratios (OR) with corresponding 95% confidence intervals (CI) and P-values, illustrated as scatter plots. The evidential threshold in MR analysis was defined as P-value < 0.004(0.05/14) according to the Bonferroni correction method. P-value < 0.05 but above the Bonferroni corrected evidential threshold was regarded as a potential association. Meanwhile, a P-value < 0.05 was also considered significant in the sensitivity analysis. The R 4.0.3 software, with “TwoSampleMR” [26] and “MR-PRESSO” [25] packages were used to process and visualize the study.

## Results

### Results of the MR study

The sample overlapping of the seven diseases GWAS dataset and UK-biobank database were as follows: coronary artery disease 0%, myocardial infarction: 73.8%, heart failure: 36.96%, atrial fibrillation: 38.4%, hypertension: 0%, stroke: 0%, and type 2 diabetes: 9.8%, respectively. Even though the sample overlapping rate of myocardial infarction is comparatively high, we have followed the sample size and timeliness priority to make the best choice as much as possible.

The numbers of SNPs that ultimately identified as IVs in different outcome datasets were 35 (type 2 diabetes), 43 (coronary artery disease, myocardial infarction, heart failure and hypertension), 44 (ischemic stroke), and 45 (atrial fibrillation) respectively. The F-statistic score of all these selected SNPs were more than 10 (coronary artery disease:57.76, myocardial infarction:57.87, heart failure:57.76, hypertension:57.77, atrial fibrillation:57.52, ischemic stroke:57.76, type 2 diabetes:58.44, respectively), indicating a low risk of weak-instrument bias.

**Fig. 1:**
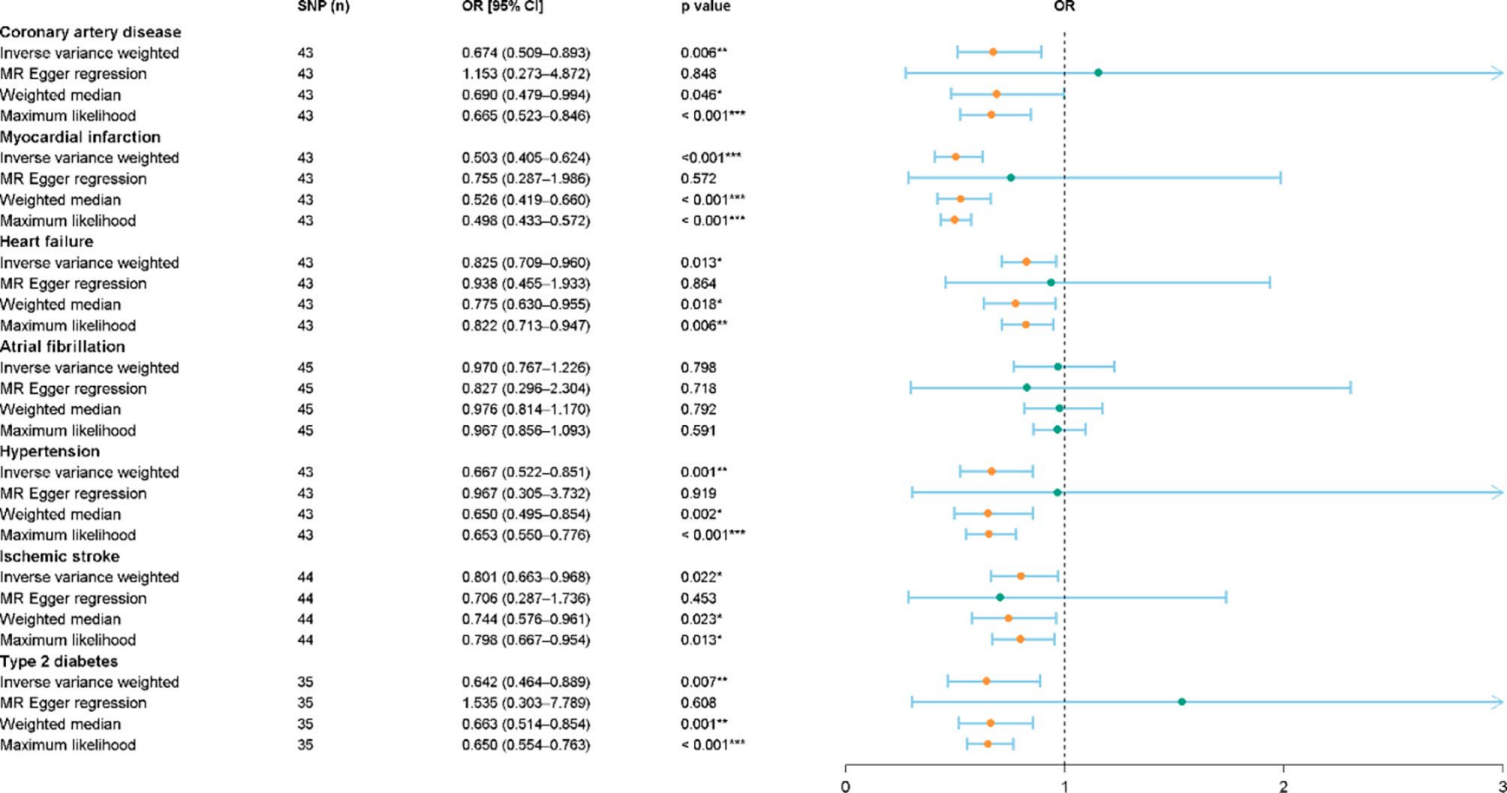
The result of MR study illustrated by forest plot Note: The causal relationship between household income status and cardiovascular-related diseases by MR study. OR = Odds Ratio, CI = Confidence Interval

According to the random-effects IVW model results, as the primary standard, higher household income tended to lower the risk of genetic susceptibility to coronary artery disease (OR: 0.674, 95% CI = 0.509–0.893, P = 0.005), myocardial infarction (OR: 0.503, 95% CI = 0.405–0.625, P < 0.001), heart failure (OR: 0.825, 95% CI = 0.709–0.960, P = 0.013), hypertension (OR: 0.667, 95% CI = 0.522–0.851, P = 0.001), type 2 diabetes (OR: 0.642, 95% CI = 0.464–0.889, P = 0.007) and ischemic stroke (OR: 0.801, 95% CI = 0.662–0.968, P = 0.022). However, no evidence was reported on the potential causal relationship between household income status and atrial fibrillation (OR: 0.970, 95% CI = 0.767–1.226, P = 0.798). In addition, the results of the weighted median and maximum likelihood estimation models supported these conclusions. Unfortunately, the MR-Egger regression model results did not show statistically significant differences. In summary, according to the Bonferroni correction standard, the MR study revealed that the higher household income population tended to have a lower risk of genetic susceptibility to myocardial infarction and hypertension. It also suggested a potentially negative relationship between coronary artery disease, heart failure, type 2 diabetes, and ischemic stroke. Detailed information is displayed in the forest map in Fig. 1 and illustrated as a scatter plot (supplement Figure-1).

### Results of sensitivity analyses in the MR study

The result of Cochran’s Q test indicated certain heterogeneity among the IVs in coronary artery disease, myocardial infarction, atrial fibrillation, hypertension, and type 2 diabetes (Table 2). Hence, the random effects IVW model was applied to minimize the effect of heterogeneity in the MR study as much as possible. More importantly, no horizontal pleiotropy was detected using the MR-Egger intercept and MR-PRESSO tests (Table 2). In addition, the “leave-one-out” method indicated that no specific SNP among the IVs significantly affected the overall result (supplement Figure-2). In general, the sensitivity analysis verified the robustness of the conclusions.

**Table 2:** The results of heterogeneity and horizontal pleiotropy tests

Diseases	Heterogeneity test		Horizontal pleiotropy test	
	MR-Egger regression	IVW model	MR-Egger intercept	MR-PRESSO test
Coronary artery disease	0.034	0.036	0.462	-
Myocardial infarction	< 0.001	< 0.001	0.402	0.988
Heart failure	0.173	0.197	0.722	0.265
Atrial fibrillation	< 0.001	< 0.001	0.755	0.685
Hypertension	< 0.001	< 0.001	0.457	0.663
Ischemic stroke	0.195	0.223	0.780	0.202
Type 2 diabetes	< 0.001	< 0.001	0.291	0.831

**Note**: IVW: Inverse Variance Weighting. P-value < 0.05 was considered as with statistical differences in both heterogeneity and horizontal pleiotropy tests

### Results of reverse MR study and sensitivity analyses

The numbers of SNPs that ultimately identified as IVs in different cardiovascular-related diseases were 115 (type 2 diabetes), 24 (coronary artery disease), 74(myocardial infarction), 9 (heart failure), 53 (hypertension), 7 (ischemic stroke), and 111 (atrial fibrillation) respectively in the reverse MR study.

**Fig. 2:**
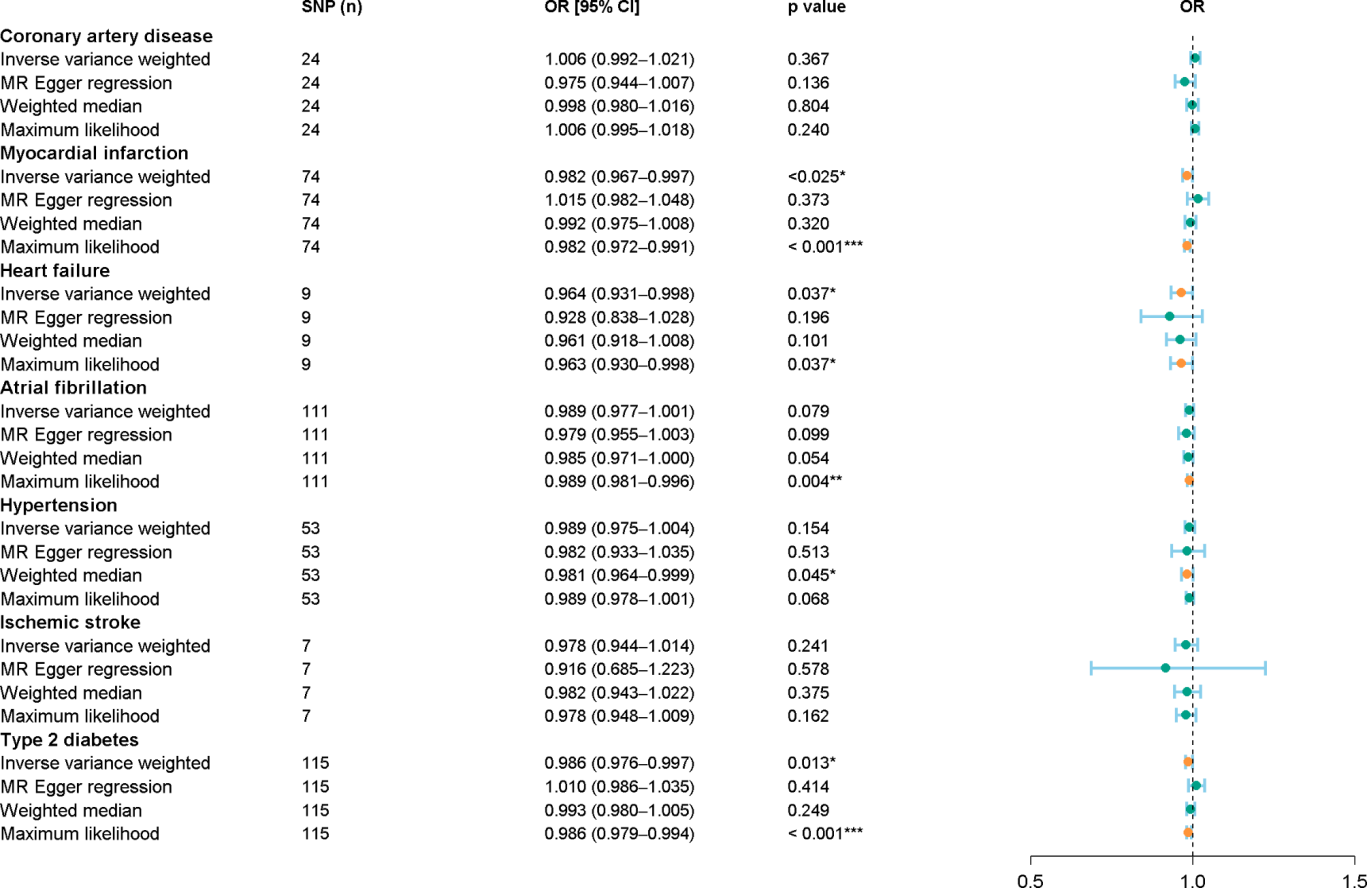
The result of reverse MR study illustrated by forest plot Note: The causal relationship between cardiovascular-related diseases and household income status by reverse MR study. OR = Odds Ratio, CI = Confidence Interval

Based on the random-effects IVW model results, there were potential indications of causal association with myocardial infarction (OR: 0.982, 95% CI = 0.967–0.997, P = 0.025), heart failure (OR: 0.964, 95% CI = 0.931–0.998, P = 0.037), type 2 diabetes (OR: 0.986, 95% CI = 0.976–0.997, P = 0.013) and household income status. The results of the maximum likelihood estimation model support these conclusions. However, the results of the MR-Egger regression model and the weighted median model did not show significant statistical differences. The detailed information is displayed in Fig. 2. However, horizontal pleiotropy was detected in SNPs of myocardial infarction (P = 0.027) and type 2 diabetes (P = 0.034) using the MR-Egger intercept test in the sensitivity analysis. This resulted in a lack of stability in the conclusions. Thus, the reverse MR study’s results only suggested a potentially negative association between heart failure and household income status.

## Discussion

Epidemiological studies have revealed that socioeconomic status has a non-negligible effect on cardiovascular health. Hence, household income status, as an indispensable part of socioeconomic status, has been consistently considered to be associated with the risk of cardiovascular disease [27]. However, in-depth causality needs further exploration. To the best of our knowledge, this research is the first that focuses on the causal effect between household income status and cardiovascular health using a bidirectional two-sample MR study. The results revealed that the population with higher household income status tended to have a lower risk of genetic susceptibility to myocardial infarction and hypertension, and suggested a potentially negative relationship with coronary artery disease, heart failure, type 2 diabetes, and ischemic stroke. Moreover, a reverse MR study indicated a potential negatively association between heart failure and household income status.

These conclusions were supported by accumulating evidence from cohort studies and meta-analyses. For example, Kucharska-Newton et al. summarized two independent cohorts from the US and Finland with 14 years of follow-up. They demonstrated that low-income status was significantly associated with an increased risk of major adverse cardiac events [28]. Similarly, in America and England, the aging population in low socioeconomic levels has been reported to have nearly double the cardiovascular disease-related mortality rates than that at superior socioeconomic levels [29, 30]. Moreover, Wang et al. conducted a study with 8,989 samples concentrated between income changes and cardiovascular disease risk. They concluded that changes in income might result in negative regulation of cardiovascular health over 17 years [31]. Additionally, it is worth noting that there is a bidirectional negative causal relationship between household income status and heart failure. On the one hand, low-income populations lack advanced medical treatment and quality care [32, 33]. Moreover, they neglect necessary health maintenance behaviors, such as annual medical checkups or adherence to secondary prevention medications. This may result in a worse prognosis of myocardial infarction and likely development of heart failure [34–36]. On the other hand, patients with heart failure have limited mobility to various degrees or disabilities in daily work that cause their income and quality of life to decline. Overall, the significant bidirectional negative causal relationship between low household income and heart failure is a complex public health concern. Therefore, policymakers for medical reimbursement should consider household income inequalities and continuously improve the fairness and accessibility to medical services for people [37].

Several relevant mechanisms which may explain the association between household income status and genetic susceptibility to cardiovascular diseases. They include: (1) low-income populations’ likelihood to consume fast foods that are more energy-dense and yield more calories for a given price [38], which creates a long-term tendency to obesity and metabolic syndromes. (2) low-income populations face greater living pressures that may increase depression and anxiety, which are ultimately linked to cardiovascular diseases [39, 40]. (3) low-income populations are more likely to face poor living environments, smoke, and engage in lifestyle behaviors such as drinking, which are already identified as risk factors for cardiovascular health [41, 42].

The biological functions of SNPs as IVs and how they affect cardiovascular health deserve further discussion. Hill et al. reported 30 independent loci associated with individual income, which may involve the biological process of GABAergic and serotonergic neurotransmission [43]. The current research indicated that dysregulation of the GABA signaling pathway was linked to psychiatric disorders, such as depression, autism, and anxiety [44, 45]. Among the 30 reported genetic loci, seven SNPs share common with the selected IVs in our study (rs11588857, rs6699397, rs32940, rs10429582, rs2332719, rs1455350, rs784256). The result enlightens us that household income status may affect the progress of cardiovascular diseases through GABA-mediated mental or psychological-related biological mechanisms. Nevertheless, more direct evidence is needed to support this assumption.

The greatest advantage of this bidirectional MR study is that our findings effectively avoided the influence of reverse causes and minimized residual confounding. However, some limitations of the study should be mentioned. First, the heterogeneity test results suggested certain heterogeneity among the IVs. Although the random effects IVW model has been applied to minimize the effect of heterogeneity in MR study as much as possible. Second, models based on different assumptions involved in MR studies have distinctive advantages and disadvantages that may increase the likelihood of obtaining inconsistent or contradictory results. Thus, this study’s results should be cautiously interpreted. Third, the sample overlapping of the myocardial infarction GWAS dataset and UK-biobank database was comparatively high, which may increase potential bias.

## Conclusion

This study explored the causal relationship between household income status and cardiovascular health using a bidirectional MR study based on datasets with million samples. The results revealed that the population with higher household income status tended to have a lower risk of genetic susceptibility to myocardial infarction and hypertension and suggested a potentially negative relationship with coronary artery disease, heart failure, type 2 diabetes, and ischemic stroke. These findings strengthen that medical reimbursement should consider household income inequalities and continuously improve the fairness and accessibility to medical services for the population in low household income status.

## Electronic supplementary material

Below is the link to the electronic supplementary material.

Supplementary Material 1

Supplementary Material 2

## Data Availability

The genetic variable information of single nucleotide polymorphisms (SNPs) was obtained from the IEU GWAS database (https://gwas.mrcieu.ac.uk/datasets/), a publicly available GWAS summary database.

## Abbreviation

MR: Mendelian Randomization
SNP: Single Nucleotide Polymorphism
GWAS: Genome-Wide Association Study
IV: Instrumental Variable
IVW: Inverse Variance Weighting
OR: Odds Ratio
CI: Confidence Interval
