# Supplementary figures and images for: Dissecting the causal relationship between household income status and genetic susceptibility to cardiovascular-related diseases: Insights from bidirectional mendelian randomization study
Source: BMC Public Health. 2023 Apr 24;23:749. doi: 10.1186/s12889-023-15561-7 (PMC10124030; doi:10.1186/s12889-023-15561-7)

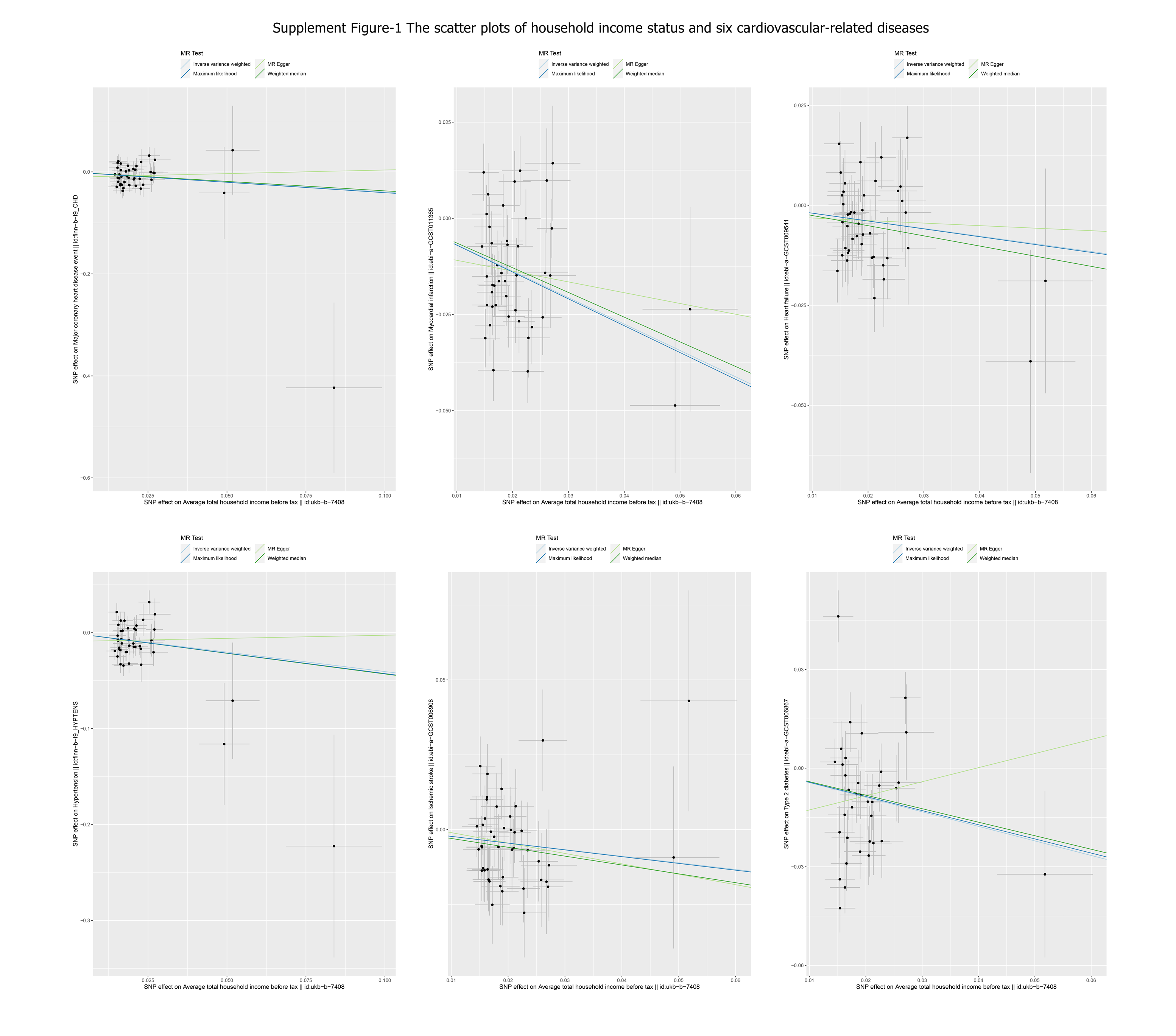

Supplement: Supplementary file 1 — Supplementary Material 1 [file 12889_2023_15561_MOESM1_ESM.png]

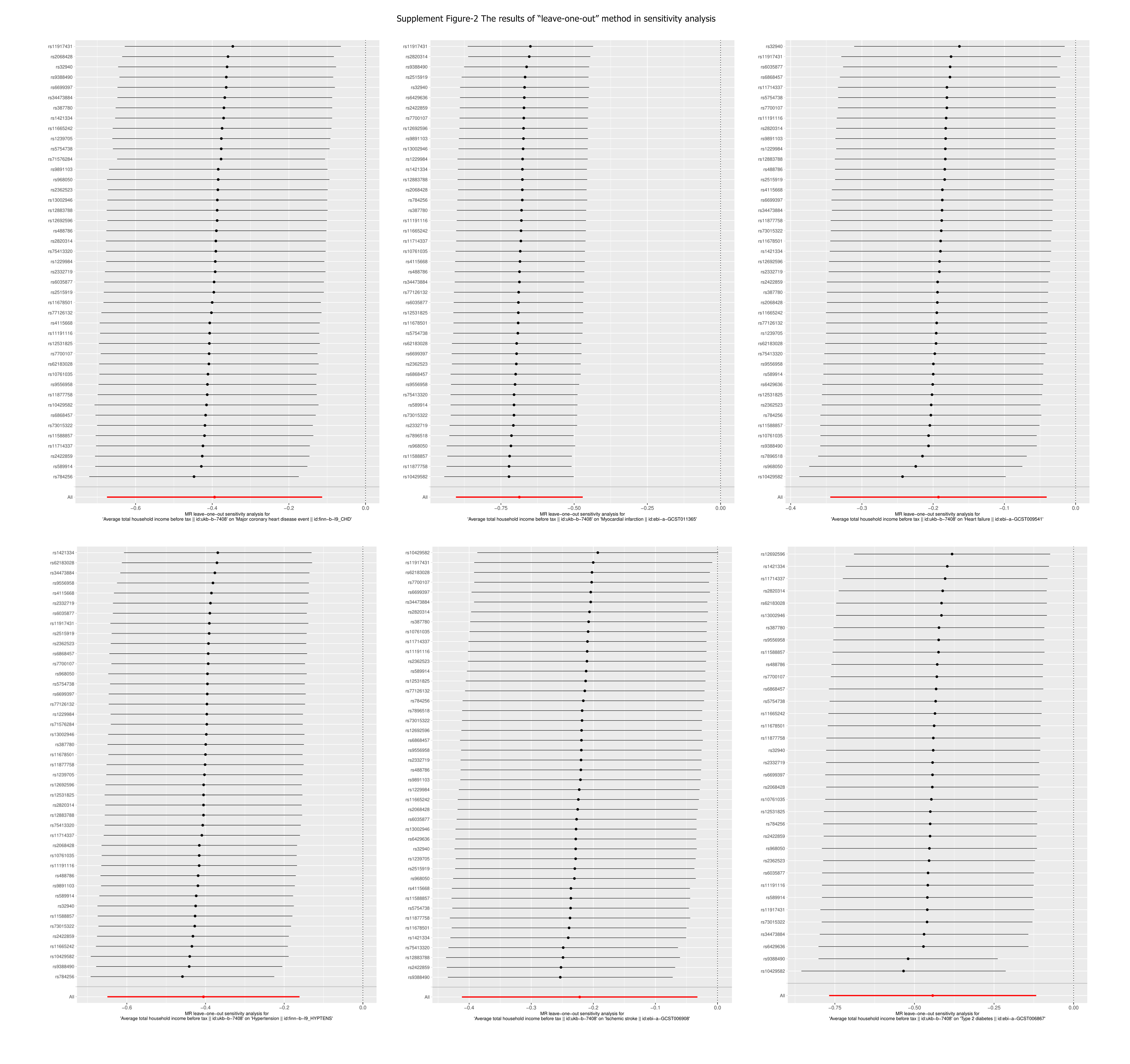

Supplement: Supplementary file 2 — Supplementary Material 2 [file 12889_2023_15561_MOESM2_ESM.png]
